# Activation of MG53 Enhances Cell Survival and Engraftment of Human Induced Pluripotent Stem Cell-Derived Cardiomyocytes in Injured Hearts

**DOI:** 10.1007/s12015-023-10596-0

**Published:** 2023-07-21

**Authors:** Ki Ho Park, Xingyu He, Lin Jiang, Hua Zhu, Jialiang Liang, Yigang Wang, Jianjie Ma

**Affiliations:** 1 Division of Surgical Sciences, Department of Surgery, University of Virginia School of Medicine, Charlottesville, VA USA; 2 Department of Pathology and Laboratory Medicine, College of Medicine, University of Cincinnati, Cincinnati, OH USA; 3 Department of Surgery, College of Medicine, The Ohio State University, Columbus, OH USA

**Keywords:** Doxycycline, MG53, Human induced pluripotent stem cells, Cardiomyocytes, Myocardial infarction, Ischemia–reperfusion, Immunocompromised mice

## Abstract

**Background and Objective:**

Our previous studies demonstrated that MG53 protein can protect the myocardium, but its use as a therapeutic is challenging due to its short half-life in blood circulation. This study aimed to investigate the cardioprotective role of MG53 on human induced pluripotent stem cell-derived cardiomyocytes (HiPSC-CMs) in the context of myocardial ischemia/reperfusion (I/R).

**Methods:**

In vitro*:* HiPSC-CMs were transfected with adenoviral MG53 (HiPSC-CMs^MG53^), in which the expression of MG53 can be controlled by doxycycline (Dox), and the cells were then exposed to H_2_O_2_ to mimic ischemia/reperfusion injury. In vivo*:* HiPSC-CMs^MG53^ were transplanted into the peri-infarct region in NSG™ mice after I/R. After surgery, mice were treated with Dox (+ Dox) to activate MG53 expression (sucrose as a control of -Dox) and then assessed by echocardiography and immunohistochemistry.

**Results:**

MG53 can be expressed in HiPSC-CM^MG53^ and released into the culture medium after adding Dox. The cell survival rate of HiPSC-CM^MG53^ was improved by Dox under the H_2_O_2_ condition. After 14 and 28 days of ischemia/reperfusion (I/R), transplanted HiPSC-CMs^MG53^ + Dox significantly improved heart function, including ejection fraction (EF) and fractional shortening (FS) in mice, compared to HiPSC-CMs^MG53^-Dox, and reduced the size of the infarction. Additionally, HiPSC-CM^MG53^ + Dox mice demonstrated significant engraftment in the myocardium as shown by staining human nuclei-positive cells. In addition, the cell survival-related AKT signaling was found to be more active in HiPSC-CM^MG53^ + Dox transplanted mice’s myocardium compared to the HiPSC-CM^MG53^-Dox group. Notably, the Dox treatment did not cause harm to other organs.

**Conclusion:**

Inducible MG53 expression is a promising approach to enhance cell survival and engraftment of HiPSC-CMs for cardiac repair.

**Graphical Abstract:**

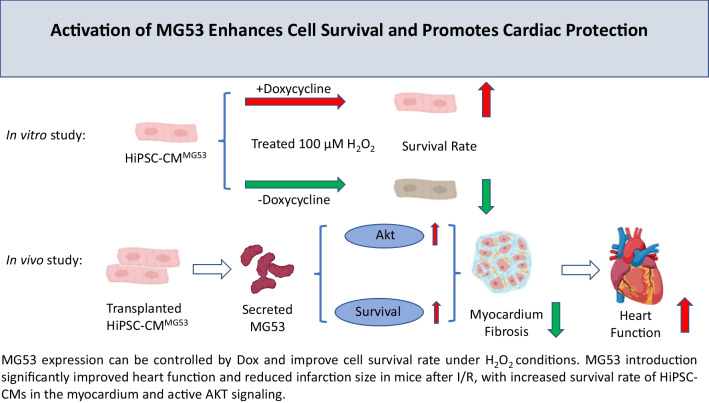

**Supplementary Information:**

The online version contains supplementary material available at 10.1007/s12015-023-10596-0.

## Introduction

Myocardial infarction (MI) continues to be a major health challenge despite significant reductions in mortality rates over recent decades [1]. Cell therapeutics are rising in use due to their potential beneficial effects. The most common route of cell delivery for myocardial therapy involves intravenous, cell sheet, or direct intramyocardial injection into infarcted hearts [2]. One alternative treatment that has shown promise in preclinical studies is the use of human induced pluripotent stem cells (HiPSCs) for infarcted myocardial repair [3]. HiPSCs can be expanded indefinitely in culture, producing a potentially unlimited source of differentiated cells including cardiomyocytes (CM) for therapeutic applications [4, 5].

HiPSC-derived CMs (HiPSC-CMs) have several potential advantages for the treatment of MI, such as being generated from a patient's own cells, allowing for a personalized treatment approach that minimizes the risk of immune rejection and reduces the need for immunosuppressive drugs [6]. Importantly, the risk of tumorigenicity of pluripotent stem cells can be avoided by morphological selection, labelling, or staining strategies using fluorescence activated cell sorters (FACS), or drug selection approaches [3, 7]. Remarkably, HiPSC-CMs can be produced consistently and reproducibly using a chemical method based on the marked glucose/lactate metabolism characteristics of CMs, with purity reaching up to 99% and avoiding tumor formation after transplantation [8], ensuring a standardized and reliable product for use in therapy. However, survival rates of injected cells are typically low, making it desirable to find a strategy that can achieve sustained cell delivery and promote engraftment of implanted cells.

HiPSC-CM can be tested for safety and efficacy before use in patients and also can be genetically modified or engineered to enhance their therapeutic potential. MG53, a protein encoded by the TRIM72 gene, is predominantly expressed in mouse skeletal and heart muscle and has been shown to protect cardiomyocytes from injury and reduce the size of myocardial infarction in animal models [9–13]. In addition to facilitating repair of injury to cardiomyocytes, MG53 also has anti-inflammation function to preserve heart function during aging [14]. As a naturally occurring protein, MG53 is unlikely to cause an immune response, making it a potentially safe and well-tolerated therapeutic agent that can be delivered systemically or locally [15, 16].

In this study, we investigated the role of MG53 in protecting implanted HiPSC-CMs in infarcted hearts. HiPSC-CMs were engineered to secrete MG53 under the control of doxycycline (Dox). We demonstrated that Dox treatment activated MG53 and improved engraftment of HiPSC-CMs in infarct hearts, resulting in the reduction of fibrotic remodeling and restoration of heart function after MI via activation of the AKT signaling pathway.

## Material and Methods

### Animal Care and Experimental Mice

Animal handling and surgical procedures were performed according to protocols approved by the Institutional Animal Care and Use Committee (IACUC) of The Ohio State University and were compliant with guidelines of the American Association for the Accreditation of Laboratory Animal Care. All experimental mice purchased from The Jackson Laboratory were 10-week-old immune deficiency (NOD scid gamma) mice (NSG™, Jackson Lab stock No: 005557) to minimize immune rejection for human cell transplantation.

### Generation of mCherry-TRE-tPA-MG53 Adenovirus

mCherry-TRE-tPA-MG53 cassettes were subcloned into pShuttle-CMV vector (Clontech, Mountain View, CA) and recombinant adenovirus genomic DNA were generated using the AdEasy-1 Adenovirus system (Agilent Technologies, La Jolla, CA) according to the manufacturer’s instructions [17]. The adenoviral particles were packaged and amplified in AD293 cells, and the viral medium was collected after 3 days transfection and stored at -80ºC for use.

### HiPSC Culture and Adenovirus Infection

The Gibco human episomal iPSC (HiPSC) line was purchased from Thermo Fisher Scientific and adapted to feeder-free culture conditions. The cell maintenance and differentiation approaches were performed according to the manufacturer’s instructions. Briefly, HiPSCs were maintained in Essential 8™ culture medium on culture dishes coated with vitronectin (0.5 µg/cm^2^) at 37 °C in a humid atmosphere of 5% CO_2_ and expanded for 3–4 days. After HiPSC reached 80–90% confluency, culture medium was changed from Essential 8™ to Gibco™ PSC Cardiomyocyte Differentiation Kit medium A (Thermo Fisher Scientific, A2921201). Adenovirus were added in medium when changing from Cardiomyocyte Differentiation medium B to Cardiomyocyte Maintenance Medium. RPMI 1640 medium supplemented with 4 mM L-lactate was used to purify the differentiated cells. This selective medium allows only cardiomyocytes to survive after a period of 96 h. After 6–8 h of adding adenovirus, the medium was changed to wash out any remaining adenovirus. After 48 h of adenovirus infection, the majority of HiPSC-CMs expressed mCherry, and beating was observed, confirming the onset of cardiomyocyte functionality. Finally, 1 μg/mL of doxycycline (Dox) was added into the cell culture to activate MG53 expression in HiPSC-CM (HiPSC-CM^MG53^).

### Cell Death and Viability Assay

After adenovirus infection, control cells or HiPSC-CM^MG53^ were treated with 100 µM H_2_O_2_ for 6 h. The viability of human induced pluripotent stem cell-derived cardiomyocytes (HiPSC-CMs) was assessed in our in vitro study using Calcein-AM from the LIVE/DEAD Viability Kit (Invitrogen, L-3224) and Hoechst 33342 (Thermo Scientific, 62249). Briefly, after subjecting the cells to their respective treatments, Hoechst 33342 was added at a concentration of 1 µg/ml to each of the four groups before incubation for 5–10 min. Following this, the medium was removed and replaced with 2 µM Calcein-AM in Dulbecco's Phosphate-Buffered Saline (DPBS), then incubated for 30 min at room temperature. The survival rate of the cells was then calculated based on the ratio of Calcein-AM positive cells (live cells) to Hoechst 33342 positive cells (total cells).

### Surgical Procedures for Mouse I/R Model Cell Transplantation

Murine I/R injury models were performed as described in the previous studies by us [18, 19]. The NSG™ mice, aged 11–12 weeks, were subjected to isoflurane anesthesia induced by spontaneous inhalation, and maintained under general anesthesia with 2% isoflurane. The inhalation gas was a mixture of oxygen (98.0%) and isoflurane (2.0%). The mice were mechanically ventilated using a rodent ventilator (Model 683, Harvard Apparatus, South Natick, MA) connected to a tracheal tube. Throughout the surgical procedure, the body temperature of the mice was maintained at 37 °C on a warm pad. A left-side limited thoracotomy was performed to expose the heart, and the left anterior descending artery was temporarily knotted using a 6–0 polyester suture placed on a piece of PE-10 tubing at 1 mm from the tip of the normally positioned left auricle. A knot measuring 2–3 mm was tied around both the LAD and PE-10 tubing to create the ligation and cause ischemic injury when the suture was tightened. Heart rate and ST elevation were monitored by an electrocardiogram. Ischemia was confirmed by ST elevation and color change of myocardium downstream of LAD for 60 min. After creating a first knot, a second slipknot was created so that the ligation could be easily removed when reperfusion was to be started. In sham-operated animals, the suture was placed beneath the LAD without ligation. To infuse HiPSC-CMs transfected by adenovirus, approximately 25 µL of cell suspension (5 × 10^5^ cells/mL) was injected into the anatomically similar region at the border zone of the infarcted area 5 min before reperfusion. The rate of infusion was controlled to 10 µL/min using an Advance™ series 1200 infusion system (CellPoint Scientific, Inc., Gaithersburg, MD). Before closing the thoracic cavity, positive end-expiratory pressure was applied to inflate the lungs. The muscle layers and skin were closed separately, allowing the animals to recover under aseptic precautions and analgesic medication (Buprenorphine, 0.1 mg/kg s.c.) to reduce post-operative pain. After recovery, mice were treated with 1% sucrose containing 2 mg/mL doxycycline or 1% sucrose only through drinking water. Excluded from further analysis were mice that died within 2 days of surgery or had no clear infarct scar.

### Echocardiography

Echocardiography was performed in mice anesthetized with 2% isoflurane, using a Vevo 2100 Imaging System (FUJIFILM Visualsonics, Inc., Toronto, Ontario, Canada) and the MX400 22 to 55 MHz ultrasound transducer. The heart was first viewed using the two-dimensional mode in the parasternal long-axis and then short-axis views. The short-axis views were used to position the M-mode cursor perpendicular to the ventricular septum and LV posterior wall. LV end-diastolic (LVDd) and end-systolic diameters (LVDs) were measured from M-mode recordings. The left ventricular ejection fraction (EF) was calculated using the formula: EF% = [(LVDd)^3^-(LVDs)^3^]/(LVDd)^3^ × 100. The left ventricular fractional shortening (FS) was determined by the formula: FS% = [(LVDd–LVDs)/LVDd] × 100. All measurements were performed according to the American Society for Echocardiography leading-edge technique standards and were averaged over three consecutive cardiac cycles.

### Histology and Immunostaining

Paraffin-embedded tissue Sects. (4 µm in thickness) were prepared for histological examination using hematoxylin and eosin (H&E) staining. Prior to fixation, tissue samples were fixed in 4% paraformaldehyde (PFA) overnight at 4 °C and washed three times for 5 min with 70% ethanol. Following fixation, the washed samples were processed and embedded in paraffin. The 4-μm-thick paraffin sections were cut into slides for pathological staining, including H&E and immunofluorescent staining. The immunofluorescent staining protocol involved deparaffinization and rehydration of the slides, which were incubated successively in xylene, 100% ethanol, 95, 75, 50% ethanol, and PBS. Antigen retrieval was achieved by heating in a pressure cooker with Tris–EDTA buffer for 13 min. The antibodies used for immunofluorescent staining were Alexa Fluor 647 Anti-Cardiac Troponin T (BD Biosciences, 565,744) and Wheat Germ Agglutinin, Alexa Fluor 488 Conjugate (Thermo Fisher Scientific, W11261). HiPSC-CMs were detected by Anti-Human Nuclear Antigen (Abcam, ab191181). Fluorescent imaging was performed with an Olympus BX41 microscope equipped with an epifluorescence attachment, and the images were analyzed using ImageJ software.

## Immunoblotting

HiPSC-CMs and heart tissue lysates were separated by 10% SDS-PAGE and transferred onto polyvinylidene fluoride membranes (Millipore). The blots were washed with Tris-buffered saline Tween-20 (TBST), blocked with 5% milk in TBST for 1 h. Amongst the different experiments, membranes were incubated with custom-made mAb-MG53, glyceraldehyde 3-phospate dehydrogenase (GAPDH; Santa Cruz, sc-32233), and anti-AKT (phospho T308) antibody (Abcam, ab38449). Different amounts of rhMG53 protein were loaded as positive controls. Immunoblots were visualized with an ECL Plus kit (Pierce). To evaluate the secretion level of MG53 from HiPSC-CMs induced by doxycycline, the samples were prepared by acetone precipitation of proteins from the collected culture medium and PBS used to wash the dish.

### Data Handling and Statistical Analysis

D’Agostino and Pearson normality test was used to evaluate normality of the data. If normal, we used unpaired t-test for single comparison and Tukey test for multiple comparisons. If not normal, we used nonparametric test (Mann–Whitney U test for single comparison or Dunn test for multiple comparisons) to derive the P. Data are represented as mean ± SEM. A value of P < 0.05 was considered significant. All data were analyzed using Excel and GraphPad Prism 9 software.

## Results

### Engineering HiPSC-CMs to Secrete MG53 in Dox Dependent Manner

**Fig. 1:**
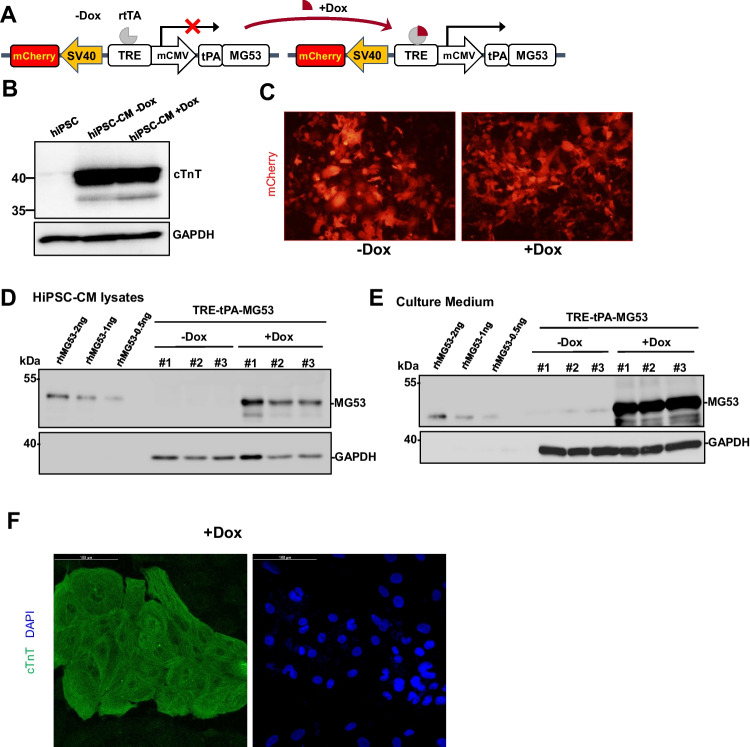
Engineering HiPSC-CM to secrete MG53 in Dox-dependent manner. **A** Adenovirus as packaged with TRE-tPA-MG53 transgene, with expression of tPA-MG53 driven by a minimum CMV promoter under the control of the tetracycline response element (TRE). This plasmid also contain the mCherry reporter cDNA driven by a separate SV40 promoter, allowing for identification of infected HiPSC-CMs. **B** Western blot reveals abundant expression of cTnT as marker of CMs. **C** Representative images show expression of mCherry as reporter gene after adenovirus infection, without Dox (-Dox) or with Dox (+ Dox) treatment. **D** MG53 protein expression induced by Dox in HiPS-CMs, detected by Western blot, #1–3 represent triplicate tests. GAPDH was used as the loading control. **E** Secretion of MG53 from HiPS-CMs induced by Dox, detected by Western blot. **F** Immunostaining of cTnT positive cells in HiPSC-CMs with Dox-induced MG53 expression

For inducible expression and secretion of MG53, the tPA-MG53 transgenes [20] were cloned behind a minimum CMV promoter under the control of the tetracycline response element (TRE) [21]. The transgene also contains the mCherry cDNA, whose expression is driven by a separate SV40 promoter (Fig. 1A). The final mCherry-TRE-tPA-MG53 construct was packaged into adenovirus (as described in Li, et. al. [17]) for infection in the HiPSCs.

HiPSCs were cultured under cardiomyocyte differentiation medium to induce formation of beating HiPSC-CMs. Western blot confirmed abundant expression of cTnT, as a marker of cardiomyocytes (Fig. 1B). After 48 h of adenovirus infection, most of the cells expressed the mCherry marker, indicating successful transfection of the gene (Fig. 1C). Doxycycline treatment at a concentration of 0.1 µg/mL did not affect the transfection efficiency. In addition, we found that HiPSC-CMs maintained beating function even at 30 days post doxycycline treatment (see Supplemental Movie).

Subsequently, we assessed the expression and secretion of MG53 in HiPSC-CMs and culture media, respectively, using Western blot analysis. Our results showed that Dox treatment (HiPSC-CM^MG53^ + Dox) induced expression of MG53 in the cell lysate (Fig. 1D), and secretion into the culture medium (Fig. 1E).

As described in our previous publication, we have a high-affinity monoclonal antibody against MG53 that allows for quantification of the MG53 protein that was inducibly upregulated by doxycycline treatment [22]. Following the same protocol, we determined that Dox-induced MG53 secretion from hiPSC-CM^MG53^ was ~ 1.6 pg/cell (Fig. 1E). Additionally, the MG53 expression did not affect the structures or purity of HiPSC-CMs after Dox induction confirmed by cTNT immunostaining (Fig. 1F). Overall, these results demonstrate the successful transfer and expression of the mCherry-TRE-tPA-MG53 gene in HiPSC-CMs, with inducible expression of MG53 by Dox treatment.

### HiPSC-CMs with Dox-Induced MG53 Secretion Protects MI in NSG™ Mice

**Fig. 2:**
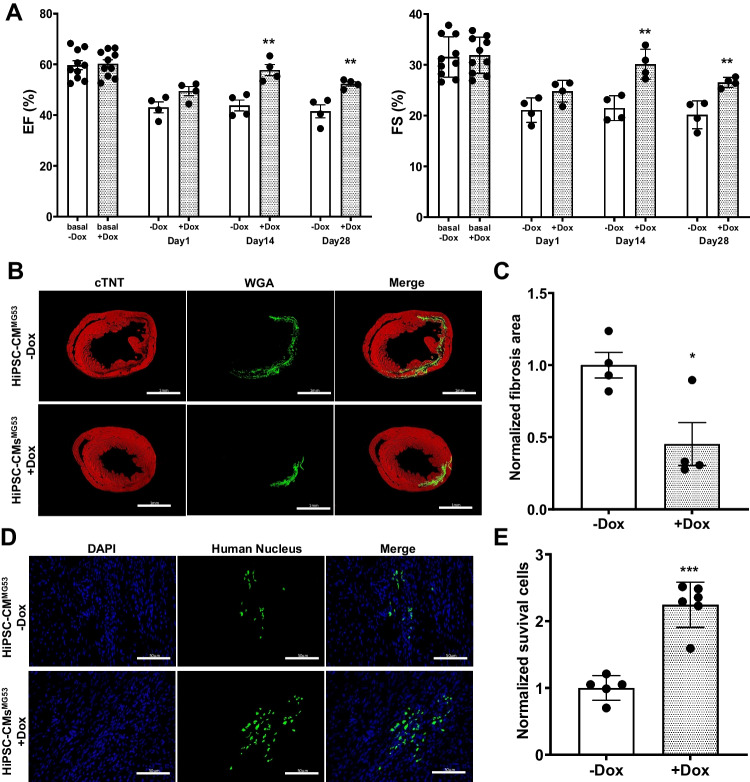
hiPSC-CMs with Dox-induced MG53 secretion protects MI in NSG mice.** A** Values of ejection fraction (EF) and fractional shortening (FS) on Day1, Day14, Day 28 post MI of NSG mice were quantified following injection of HiPS-CMs.^MG53^ with Dox (+ Dox) or without Dox treatment (-Dox). **B** Fibrosis area detected by WGA staining at Day 28 after IR surgery. **C** Statistical image analysis of fibrosis area. **D** Immunostaining of anti-human nuclear antigen (green fluorescence) to quantify the survival of HiPS-CMs at 28 days post MI without (top) or with Dox treatment (bottom). **E** Quantitative analyses showed significant increase in survival of HiPS-CMs following treatment of Dox. *P < 0.05, **P < 0.005, ***P < 0.0001

Therapeutic effects of HiPSC-CM^MG53^ were assessed after injection in the infarct boarder zones of the NSG™ mice that were subjected to MI. Heart function was evaluated through echocardiogram 1 day before and 1 day, 14, and 28 days after MI surgeries. Echocardiography results obtained 1 day after MI surgeries indicated that there was a comparable decline in EF and FS for both the HiPSC-CM^MG53^-Dox and HiPSC-CM^MG53^ + Dox groups. The values of EF and FS were significantly improved in the + Dox group compared to the -Dox group (Fig. 2A).

Additionally, we assayed the fibrosis area using WGA staining and survival of HiPSC-CMs^MG53^ at Day 28 after MI surgery [23]. The HiPSC-CM^MG53^ + Dox group showed a significantly reduced fibrosis area (Fig. 2B, C) and an increased survival rate of injected HiPSC-CMs^MG53^ + Dox (Fig. 2D, E) compared to the hiPSC-CM^MG53^-Dox group. These data suggested that doxycycline-treated MI mice expressing secreted MG53 exhibit a significant reduction in fibrosis area and a higher rate of survival in HiPSC-CMs within the heart.

### Survival Signal Pathway and Toxicity

**Fig. 3:**
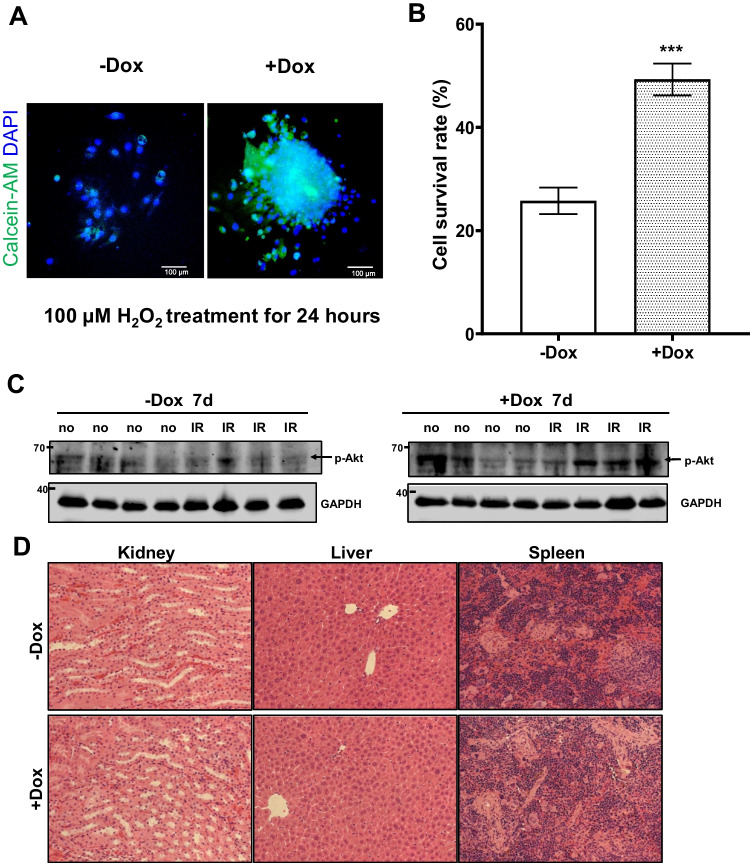
Assessment of survival signal and toxicity associated with Dox-induced MG53 activation in vitro or in vivo*.* **A** Representative images of Calcein-AM positive HiPSC-CMs after 24 h treatment of 100 µM H_2_O_2_ (in vitro). HiPSC-CMs were induced to express MG53 (+ Dox, -Dox as control) (Green: Calcein-AM live cells, Blue: DAPI stained nucleus). **B** Quantitation of cell survival rate after treatment of 100 µM H_2_O_2_ (in vitro). ***P < 0.001. **C** Western blotting of AKT survival signal pathway in the infarcted hearts implanted with HiPSC-CMs (in vivo). (no—no risk area, IR—risk area of infarction). **D** H&E staining of multi-organs with or without Dox treatment in mice injected with HiPSC-CMs at 28 days post IR injury (in vivo)

The protective effect of MG53 on HiPSC-CMs were tested using 100 µM H_2_O_2_ that can induce death of the cells to mimic reperfusion-related oxidative stress. The impact of Dox-induced MG53 expression on the survival of HiPSC-CMs^MG53^ were examined in vitro. The results of viability assay demonstrated a significant increase in the survival of hiPSC-CMs^MG53^ induced with MG53 (Fig. 3A, B).

For in vivo studies, since HiPSC-CMs^MG53^ + Dox showed significant survival in the MI area due to IR (Fig. 2D, E), we investigated the expression of p-AKT, a survival signaling pathway related to MG53 as previously reported [24, 25]. The results confirmed an increase in p-AKT expression in the myocardial infarction area (Fig. 3C). Moreover, Dox-induced MG53 secretion from the implanted HiPSC-CMs^MG53^ produced no measurable toxicity in the NSG™ mice, as revealed by H/E staining of the kidney, liver, and spleen (Fig. 3D). Additionally, we did not detect any teratoma formation during the 28-day monitoring period.

## Discussion

Stem cell therapeutics utilizing HiPSC-CMs have attracted attention recently in the field of cardiovascular disease treatment. However, due to the low engraftment rate and viability of HiPSC-CMs in the harsh local physiological environments, the therapeutic efficacy is limited, complicating the transition into clinical studies [26, 27]. rhMG53 has been shown to have therapeutic efficacy for several organ injuries including lung, kidney, heart, and liver [11, 28–30]. However, due to the short half-life of rhMG53 in circulation, multiple injections are required for systemic delivery of rhMG53, which may result in relatively low therapeutic efficacy [15, 16]. To address these issues, a novel therapeutic approach was established for MI using a HiPSC-based platform with inducible expression of MG53. Specifically, we induced the expression of MG53 in HiPSC-CMs using Dox treatment to generate a localized and sustainable MG53 delivery to the infracted area. By engineering HiPSCs to express MG53, the cells can secrete the protein continuously, thereby improving its therapeutic efficacy and prolonging the treatment period compared with traditional systematic approaches.

For high efficiency infection of the HiPSC-CMs and inducible control of MG53 expression and secretion, the tet-on system was utilized. This system offers several advantages, including the ability to easily control MG53 expression by administering Dox. Furthermore, the tetracycline-regulated promoter system is relatively specific and typically does not induce the expression of endogenous genes. As a result, the risk of off-target effects is reduced, and any observed effects are more likely to be specifically due to MG53 expression. Additionally, we delivered the TRE-tPA-MG53 via adenovirus, which our result showed provided a high transduction efficiency.

Our results demonstrate that we have successfully engineered an HiPSCs platform to secrete MG53 in Dox dependent manner. By adding Dox, we have also demonstrated the controllability of MG53 secretion. In the in vivo experiment section, immunocompromised mice (NSG™) were used for the therapeutic evaluation of MG53-coded HiPSC-CMs. Since the NSG™ mice have deficient immune systems, there could be a reduced risk of immune rejection of the engrafted HiPSC-CMs. After the injection of HiPSC-CMs^MG53^ in the infarcted area, our results indicate that HiPSC-CMs^MG53^ secreting MG53 under Dox induction can significantly and effectively preserve heart function compared to groups without Dox induction. After 14 and 28 days of surgery, the EF value for the HiPSC-CMs^MG53^ + Dox group was relatively close to the value before surgery was performed. The results suggest that the use of MG53 could be a promising therapeutic approach for preserving heart function after MI.

WBA staining images showed that the fibrosis area was significantly reduced at Day 28 after I/R surgery, when compared with the -Dox group. This demonstrated that Dox-induced MG53 could decrease the amount of scar tissue formation, further preserving heart contraction. In addition, reducing heart fibrosis area potentially improves the electrical conduction of the heart, which could reduce the possibility of arrhythmias and other cardiac problems by avoiding electrical signal disruption. We also demonstrated that the HiPSC-CM + Dox group exhibited significantly higher cell survival rate than the HiPSC-CM-Dox group. These results further demonstrate that MG53 not only improves the viability of transplanted HiPSC-CMs in the MI area, but also provides a sustainable long-term therapeutic effect for MI patients. Transplanted HiPSC-CMs enhance heart contractile force through external mechanical support, while secreted MG53 provides local or systematic treatment to preserve heart function.

AKT activation can promote cell survival by inhibiting apoptosis [31, 32]. In our previous research, the interaction between MG53 (a protein involved in cell membrane repair and stress response) and insulin receptor substrate-1 (IRS-1) (a key component of the insulin signaling pathway) was investigated. The findings suggested that MG53 can bind to IRS-1, potentially leading to the activation of the PI3K/Akt pathway, a critical signal transduction pathway that promotes cell survival and growth [13, 24]. In addition to inhibiting apoptosis, AKT activation can also downregulate several proteins involved in oxidative stress and inflammation (such as p53 and NF-κB [25]) which could contribute to the higher levels of HiPSC-CM + Dox survival rate than with the -Dox group. AKT activation can enhance angiogenesis by promoting the growth and proliferation of endothelial cells through various signaling pathways. This mechanism improves the supply of oxygen and nutrients to the heart muscles, which is critical for forming new blood vessels and promoting the healing and regeneration of damaged tissue. This is particularly important in the context of myocardial infarction (MI), where reduced blood flow can lead to tissue damage and cell death. The harsh environment of the infarcted area, with low oxygen and nutrient levels, can make it difficult for cells to survive. However, AKT activation can help to promote angiogenesis and tissue regeneration in this environment, thereby improving the overall outcome.

Our study demonstrated that an hiPSC-CM-based treatment system has high potential for translational clinical regenerative medicine applications, as it did not induce any organ toxicity or inflammation. The benefits of AKT activation (including promotion of cell survival and reduction of oxidative stress and inflammation) suggest that the use of MG53 and activation of the AKT signaling pathway may be a promising therapeutic strategy for preserving heart function and promoting recovery after MI and I/R injury. These findings have significant implications for the development of novel treatments and therapeutics designed for patients with cardiovascular diseases. Although no teratomas were observed during this period in our animal study following iPSC-CM injection, it is important to note that long-term monitoring of the engrafted cells is necessary to ensure their safety, warranting further investigation.

In summary, engineered HiPSC-CMs^MG53^ offer a promising approach to improve the therapeutic efficacy of MG53 for the treatment of MI injury. The use of the doxycycline (tet-on) system to regulate gene expression offers advantages over traditional viral-based approaches.

### Supplementary Information

Below is the link to the electronic supplementary material.

Supplementary file1 (PPTX 23046 KB)

## Data Availability

Not applicable.
