# Supplementary material for: Activation of MG53 Enhances Cell Survival and Engraftment of Human Induced Pluripotent Stem Cell-Derived Cardiomyocytes in Injured Hearts
Source: Stem Cell Rev Rep. 2023 Jul 21;19(7):2420–8. doi: 10.1007/s12015-023-10596-0 (PMC10579131; doi:10.1007/s12015-023-10596-0)
Supplement: Supplementary file 1 — Supplementary file1 (PPTX 23046 KB) [file 12015_2023_10596_MOESM1_ESM.pptx]

## Slide 1
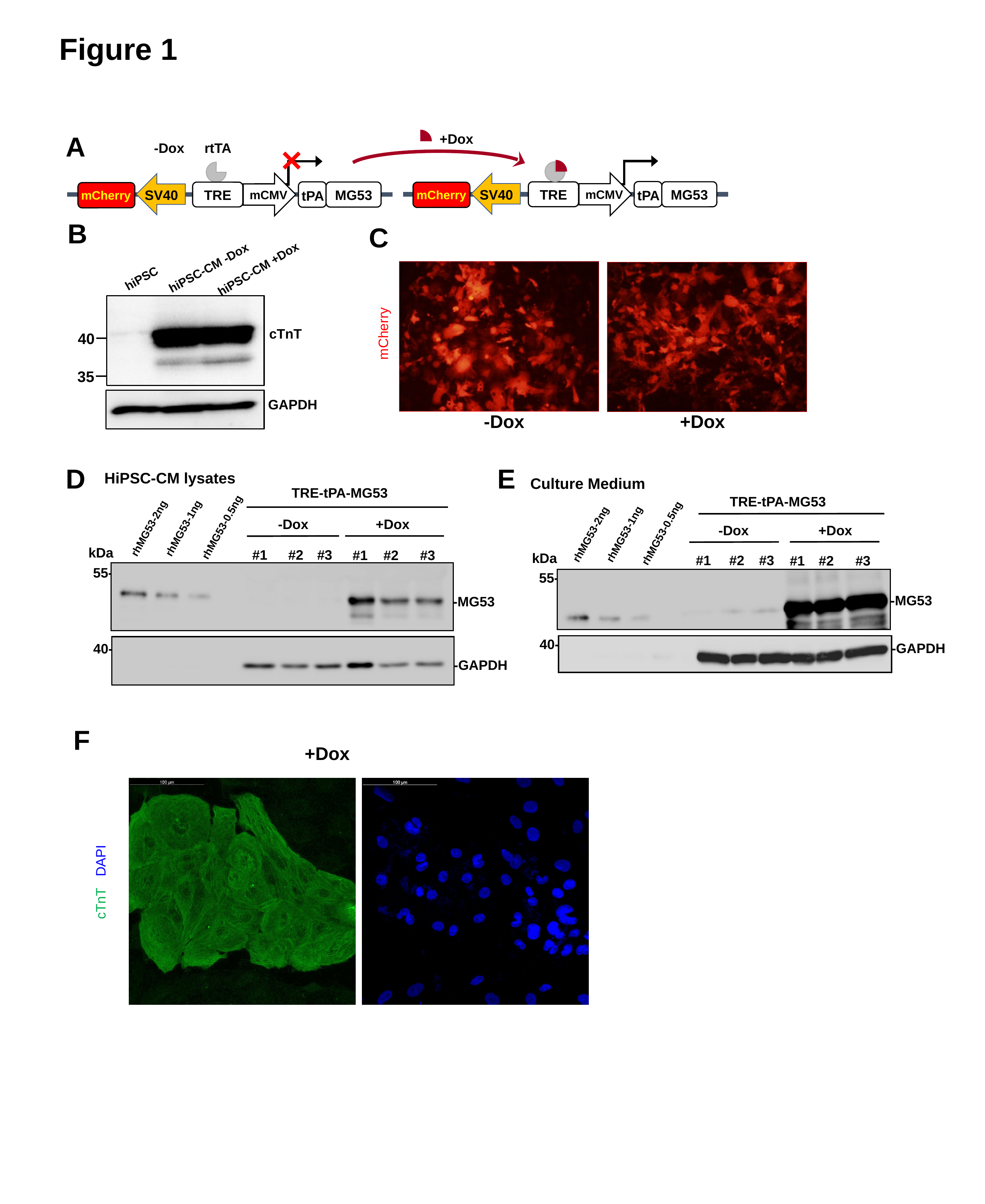

Figure 1
A
+Dox
-Dox
rtTA
mCMV
tPA
MG53
TRE
mCMV
tPA
MG53
TRE
SV40
mCherry
SV40
mCherry
B
C
hiPSC-CM -Dox
hiPSC-CM +Dox
hiPSC
40
35
cTnT
GAPDH
mCherry
-Dox
+Dox
D
E
HiPSC-CM lysates
Culture Medium
TRE-tPA-MG53
-Dox
+Dox
rhMG53-0.5ng
rhMG53-2ng
rhMG53-1ng
kDa
#1
#2
#3
#1
#2
#3
55-
-MG53
40-
-GAPDH
TRE-tPA-MG53
-Dox
+Dox
rhMG53-0.5ng
rhMG53-2ng
rhMG53-1ng
kDa
#1
#2
#3
#1
#2
#3
55-
-MG53
40-
-GAPDH
F
+Dox
cTnT DAPI

## Slide 2
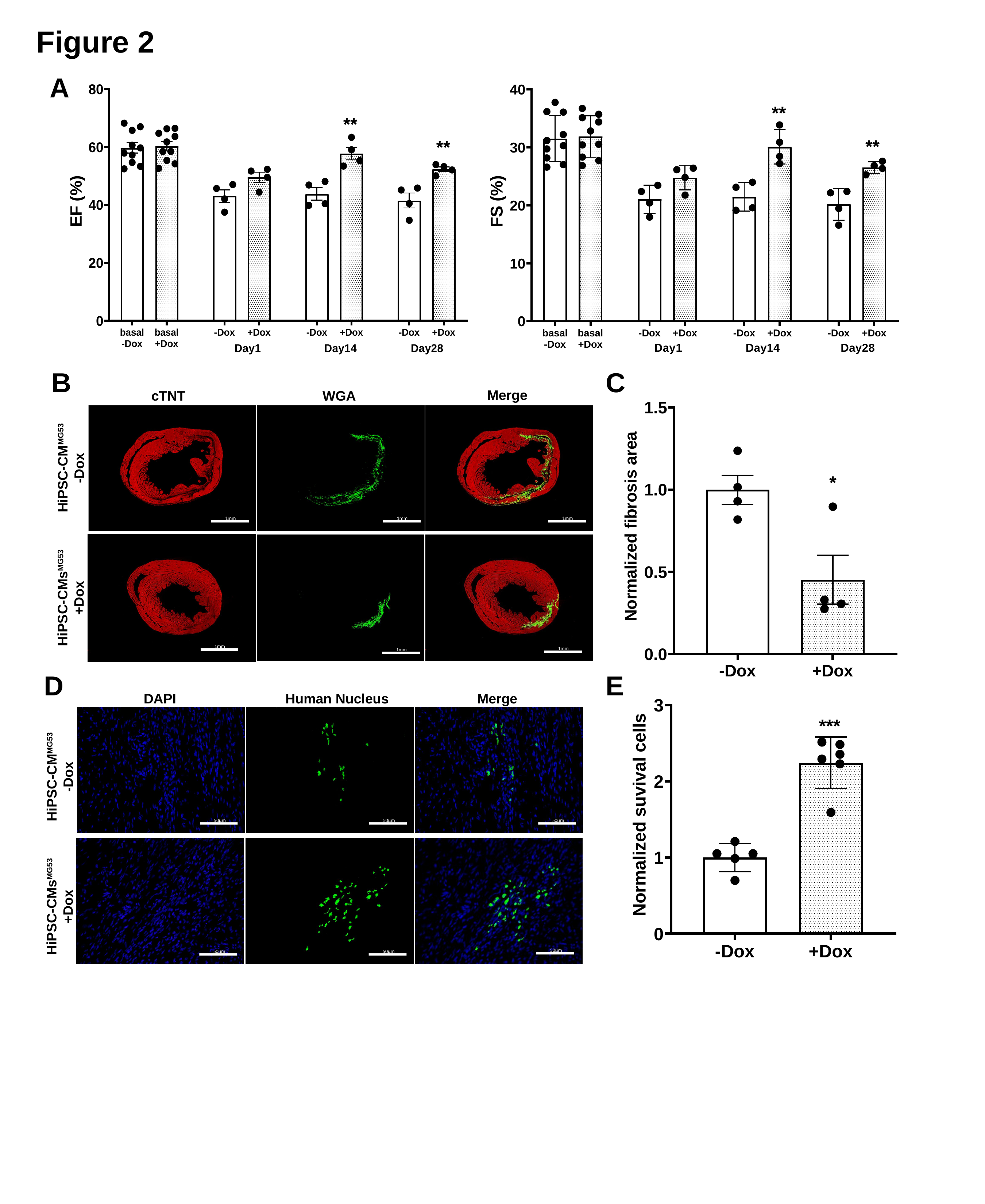

Figure 2
A
**
**
**
**
C
B
Merge
cTNT
WGA
HiPSC-CMMG53
-Dox
1mm
1mm
1mm
HiPSC-CMsMG53
+Dox
1mm
1mm
1mm
*
D
E
***
Human Nucleus
DAPI
Merge
50µm
50µm
50µm
50µm
50µm
50µm
HiPSC-CMMG53
-Dox
HiPSC-CMsMG53
+Dox

## Slide 3
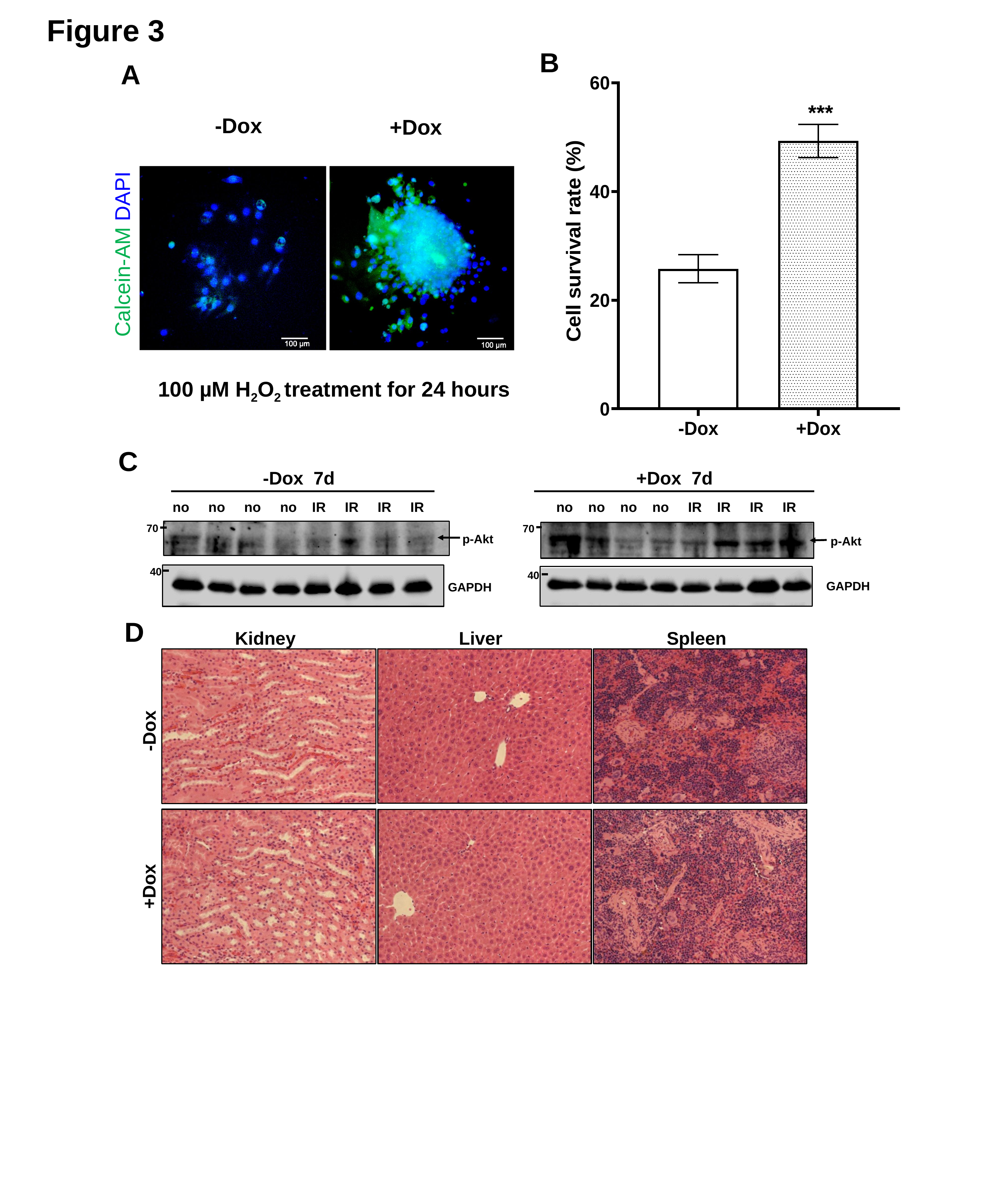

Figure 3
B
***
A
-Dox
+Dox
Calcein-AM DAPI
100 µM H2O2 treatment for 24 hours
C
-Dox 7d
+Dox 7d
no no no no IR IR IR IR
no no no no IR IR IR IR
70
70
p-Akt
p-Akt
40
40
GAPDH
GAPDH
D
Kidney
Liver
Spleen
-Dox
+Dox

## Slide 4
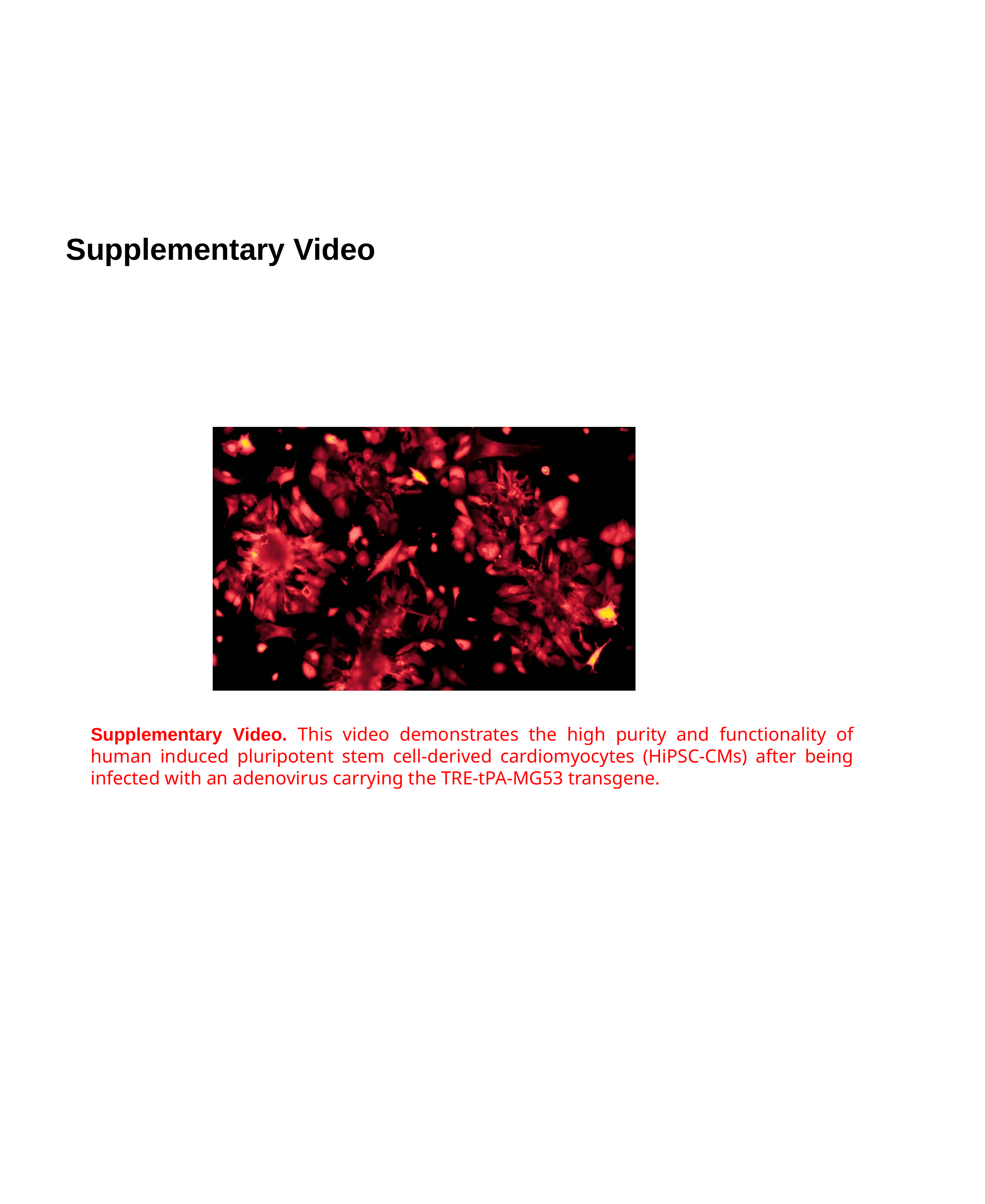

Supplementary Video
Supplementary Video. This video demonstrates the high purity and functionality of human induced pluripotent stem cell-derived cardiomyocytes (HiPSC-CMs) after being infected with an adenovirus carrying the TRE-tPA-MG53 transgene.
